# An Integrative Review to Examine the Care Pathways and Support Available for Individuals Diagnosed with Lung Cancer Who Have Never Smoked

**DOI:** 10.3390/curroncol33010004

**Published:** 2025-12-20

**Authors:** Christopher Dodd, Catherine Henshall, Mohini Jain, Zoe Davey

**Affiliations:** 1 Oxford Institute of Applied Health Research, Oxford Brookes University, Oxford OX3 0BP, UK; chenshall@brookes.ac.uk (C.H.); jainmohini64@gmail.com (M.J.); 2 Oxford Health NHS Foundation Trust, The Warneford Hospital, Oxford OX3 7JX, UK

**Keywords:** lung cancer, supportive care, psychooncology

## Abstract

As the proportion of people diagnosed with lung cancer who have never smoked rises, it is important to understand how their experiences differ from those of smokers. A better understanding of their experiences, views, and informational and supportive care needs is essential to ensuring an optimised patient-centred care pathway leading to improvements in patient satisfaction, quality of life, and treatment outcomes. This integrative review of the international literature identified 5866 articles by searching four academic databases, the grey literature, and hand-searching the reference lists of relevant systematic reviews. After screening, ten studies were selected for inclusion in the review. Thematic analysis identified five themes that spoke to the experiences of never smokers with lung cancer: stigma, awareness, diagnosis, the emotional response, and support. Stigma pervades, with potentially significant psychological and social consequences, negatively affecting patients emotionally and potentially delaying their diagnosis. Increasing awareness amongst healthcare professionals and the general public has the potential to reduce stigma and encourage earlier diagnosis. Support specifically tailored for never smokers with lung cancer can improve individuals’ experiences of care. The experiences of never smokers with lung cancer are unique, and more research is required to better tailor support and guidance for this cohort.

## Simple Summary

An increasing proportion of those diagnosed with lung cancer have never smoked. Commonly, these are oncogene-driven forms of lung cancer, including those with ALK, EGFR, and ROS1 mutations. This integrative review of the international literature provides evidence of never smokers’ experiences of the care pathway and the support available to them. It identifies key areas where their experiences are distinct from those of lung cancer patients with a history of smoking, exploring the impact of stigma and the potential for significant psychological and social consequences associated with a lung cancer diagnosis. This review highlights areas of the diagnostic process that are unique to this cohort, addresses the issue of awareness of lung cancer in never smokers, and identifies differences in the emotional burden they bear. Finally, it provides evidence of the benefit of targeted support for these patients. This review draws important conclusions about the needs and experiences of patients diagnosed with lung cancer who have never smoked and, crucially, also provides the basis for more research in this area.

## 1. Introduction

Lung cancer is one of the most common forms of cancer and the leading cause of cancer deaths worldwide [1]. Despite the potentially life-giving benefits of treatment for lung cancer, there is often a considerable physical and emotional burden for patients, their families, and their carers, as well as significant lifestyle changes affecting quality of life [2]. Whilst smoking remains the most common cause of lung cancer [1], an increasing proportion of people diagnosed with lung cancer report having never smoked [3,4]. The demography of this group differs from that of people diagnosed with lung cancer who have previously smoked. Epidemiological data indicates that the incidence is significantly higher in females and that patients who are diagnosed with lung cancer at a younger age are more likely to have never smoked [5,6]. The psychological and social consequences of the experiences of individuals who have never smoked can often differ from those of smokers [6,7]. The importance of supportive cancer care for lung cancer patients’ health outcomes and quality of life has been established [8], but it is essential to learn more about the needs and experiences of never smokers in this context. This can be used to inform guidelines for healthcare professionals and support mechanisms for patients, their families, and their carers.

People who have never smoked are most commonly defined as those who have smoked fewer than 100 cigarettes in their lifetime [6]. Lung cancer in this population can be caused by a range of other factors, such as occupational exposure to carcinogens, as well as exposure to radon or X-ray radiation, passive smoking, or outdoor pollution [6]. Lung cancer in those who have never smoked can also be caused by genetic alterations. A significant number of patients who are epidermal growth factor receptor (EGFR)-positive, anaplastic lymphoma kinase (ALK)-positive, or have fusions involving the ROS proto-oncogene 1 (ROS1) have never smoked. This is increasingly leading to a histological and epidemiological distinction to be drawn with that of lung cancer in those who have smoked [7].

The focus on smoking cessation as the most effective method of reducing the incidence of lung cancer has led to many individuals who have never smoked being unaware that they are also at risk of developing lung cancer and has contributed to a culture of blame associated with a lung cancer diagnosis [6]. The destructive nature of disease-related stigma is not exclusive to lung cancer, and its potential to worsen outcomes for individuals living with HIV/AIDS and liver disease has been previously identified [9,10]. Goffman (1963) defines stigma as reducing the individual to less than the sum of their parts, defining them by an attribute to which a stereotype can be applied [11]. Stigma is pervasive amongst individuals with a lung cancer diagnosis and has been found to contribute to late presentation, lower quality of life, higher levels of depression, and a lower inclination to access support [12]. This justifies the further study of how stigma and associated behaviours are experienced by those who have never smoked [6,7].

A body of evidence is growing around the different treatment needs of never smokers with lung cancer, as well as the potential for different approaches to screening based on causes besides smoking [13,14]. It is, therefore, also timely to consider the needs and experiences of never smokers in terms of the care pathway and the support available to them. The aim of this integrative review of the international literature was to examine any commonalities and variations in care pathways, support, and experiences of individuals with a lung cancer diagnosis who have never smoked. This informs conclusions about the specific needs of this cohort, which can inform clinical practice and provide the underpinnings for future research. This review was registered with PROSPERO (CRD42025650981).

## 2. Materials and Methods

An integrative review methodology, using the broad research question outlined above, was adopted in anticipation of the limited literature available in this area [15]. This allowed researchers to include all relevant primary research irrespective of study design, language, or publication status. The Preferred Reporting Items for Systematic Reviews and Meta-Analyses (PRISMA) was adhered to [16] (File S3).

The relevant literature was identified through a search of the PubMed, Web of Science, CINAHL, and PsychInfo databases in February 2025. A combination of MeSH and search terms was used to identify research that captured the experiences of, and support available to, individuals with a lung cancer diagnosis who have never smoked. Search terms were identified through a comprehensive consultation with the background literature and in consultation between all reviewers. Table 1 provides the PubMed search as an example of the search strategy. The full search strategy has been provided as supplementary material (File S1). 

Articles were included if they contained data that described the care pathway or the guidance or support available to people with a lung cancer diagnosis who have never smoked. This included data describing initiatives, support groups, and counselling that are exclusive to this cohort or findings that explore the patient experience of the pathway or support that is provided. For the purposes of this review, people who have smoked fewer than 100 cigarettes in their lifetime are classified as having never smoked, a definition widely supported in the vast majority of the literature [6], although studies that kept to this definition in principle were included, as well as those that did not state the definition used. Mixed population studies were included only if the data related to those who have never smoked could be identified separately from other participants, or if the proportion of participants who have never smoked was 50% or more.

In keeping with the integrative review methodology, all articles that contained primary data were included in this review. Systematic and literature reviews were excluded, but their reference lists were hand searched, as were the reference lists of included studies. Google and Google Scholar were used to identify the grey literature, and the first 50 search results from each were considered for inclusion. Furthermore, websites of 37 international organisations that provide guidance, support, and information for individuals with lung cancer were hand-searched to identify the relevant literature.

**Figure 1:**
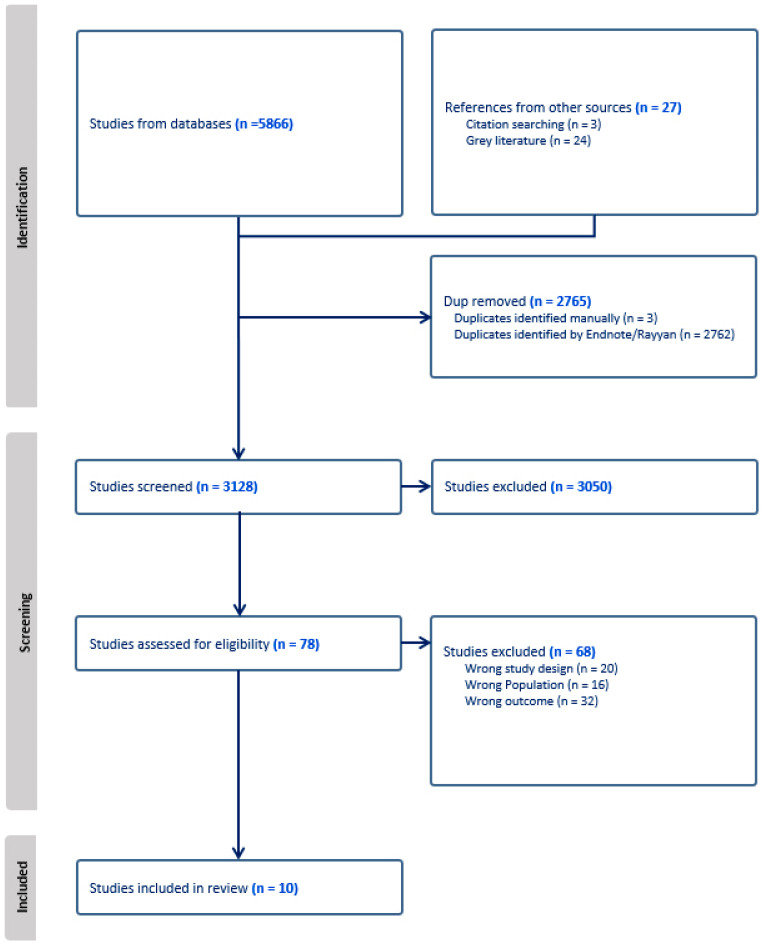
PRIMSA flow diagram of the search strategy.

The search results were imported into Rayyan, duplicates were removed, and a minimum of two reviewers screened the titles and abstracts of 3104 studies, selecting 73 articles for full-text review. Any conflicts were resolved through a discussion between the full review team. Full texts were double-reviewed, and any conflicts were resolved between the whole review team, leaving ten papers selected for inclusion in the review. Figure 1 is a visual representation of the study identification process.

Data were extracted by two reviewers (C.D. and M.J.) and tabulated into a bespoke table. Consensus was reached through discussion with the full review team. A narrative synthesis [17] was employed by conducting a thematic analysis of the tabulated data. A meta-analysis of the quantitative data was deemed inappropriate due to the heterogeneity of the studies and data. Joanna Briggs Institute critical appraisal tools were utilised by two reviewers independently (C.D. and M.J.) to assess the quality of the included studies [18,19].

## 3. Results

Of the ten studies selected for inclusion in the review, six were of a qualitative design [20,21,22,23,24,25] and four were quantitative [26,27,28,29]. Table 2 provides the details of each of the studies included in the review. Three of the qualitative studies used semi-structured interviews, and one used unstructured interviews for data collection. The other two qualitative studies used cross-sectional surveys. One of the qualitative studies also engaged in consensus work to discuss the survey results with participants. Three of the quantitative studies used cross-sectional surveys for data collection, and the other used a longitudinal survey. Three studies were conducted in the UK, and another, whilst not limited to UK participants, was conducted by a charity based in the UK. Five studies were based in the USA, and one recruited participants from across Europe. All studies had individuals with lung cancer as participants, with one also including healthcare professionals and another a broad range of stakeholders. The adoption of an integrative review methodology increased the number of studies included in this review, and two studies are not from the peer-reviewed literature, one a Ph.D. thesis and the other a report published by *Lung Cancer Europe*. All of the included studies were deemed to be of sufficient quality, with no concerns about the validity of the results. The quality assessment table has been provided as a supplementary material (File S2). 

Thematic analysis of the extracted data identified five interrelated themes that speak to the experiences of individuals with lung cancer who have never smoked. The experience of stigma is particularly pervasive, cutting across the four other themes as follows:

1. Stigma;
2. Awareness;
3. Diagnosis;
4. The emotional response;
5. Support.

### 3.1. Stigma

The stigma associated with a lung cancer diagnosis was explicitly referenced in seven of the studies included in this review [20,21,22,24,26,27,28].

Three quantitative studies used validated scales to measure the different levels of stigma experienced by current, former, and never smokers: the Cancer Responsibility and Regret Scale [26,27] and the Lung Cancer Stigma Inventory [28]. Williamson et al. (2020) and Criswell et al. (2016) found that depressive symptoms were significantly associated with higher overall lung cancer stigma scores, irrespective of smoking status [26,28]. Williamson et al. (2018) discovered that declining emotional well-being was significantly associated with higher overall lung cancer stigma across a twelve-week period [27]. Never smokers did score significantly lower for internalised stigma than current and former smokers [26,27,28]. However, Criswell et al. (2016) [26] analysed specific elements of the stigma scale—personal responsibility and regret—and discovered that associations with depressive symptoms were significantly higher with those who have never smoked than with current and former smokers.

The qualitative studies identified in this review confirmed that whilst the stigma related to a lung cancer diagnosis is not unique to those who have never smoked, these individuals experience stigma in ways that are distinct from the experiences of individuals with a current or previous smoking history. Despite not engaging in what they perceive as stigmatised lifestyle behaviours themselves, they reported that their interactions with others were detrimentally impacted by perceived negative associations between smoking and lung cancer [20,21,22,24]. Never smokers felt that they were being judged negatively by others and were made to feel ashamed [22]. Participants also reported feeling that they received less support because of their lung cancer diagnosis, expressing that people with other types of cancer, such as breast cancer, were not subject to the same stigma and were treated more favourably [20].

The impact of experiencing various forms of smoking-related stigma on never smokers with lung cancer is encapsulated by a sense that their specific needs are often minimised or forgotten due to the dominance of smoking as a primary risk factor in people’s understanding of lung cancer [20,21,22]. Never smokers also described being defensive about their smoking status [20,21,22]. It was common for individuals to experience a desire to explain to others that they were not responsible for their lung cancer and to feel that they were being unfairly categorised because of the diagnosis of lung cancer [20,21].

Conversely, never smokers with lung cancer can also feel that they are being pitied by others once it is revealed that they do not have a history of smoking. This reaction has been described as equally unwelcome [20]. Never smokers were just as likely as smokers to not disclose their lung cancer diagnosis to others [27,28]. Never smokers were also just as likely as former smokers to observe stigma affecting the way they were treated by medical professionals and the association between medical stigma and depressive symptoms, distress, satisfaction with healthcare, and avoidance coping (a mechanism adopted by some patients which involves ignoring or denying the problem to reduce stress and negative emotions) were all significantly greater in never smokers [26].

### 3.2. Awareness

The need for greater awareness about the incidence and causes of lung cancer in never smokers has been highlighted as a priority for both the public and healthcare professionals. Findings from Khan et al. (2023) suggested that prioritising research and raising awareness about the other factors that can cause lung cancer, such as exposure to Radon, passive smoking, or genetic drivers, could help to combat the negative effects of stigma and improve the care pathway for patients [24]. Never smokers with lung cancer were sensitive to public perceptions associating the disease with smoking and believed that there was a lack of public awareness around the other possible causes of lung cancer and that it can affect anyone [24]. Stakeholders, including patients, healthcare professionals, academics, and charity representatives, felt that developing a better understanding of the external causes of lung cancer beyond so-called lifestyle choices, such as smoking, may help to allay feelings of anger, fear, and anxiety [24]. Stakeholders also felt there was a need to develop educational interventions for healthcare professionals to improve knowledge about lung cancer in patients who have never smoked to drive more timely diagnosis, citing studies that have already been conducted that lead to increased early identification of lung cancer [24]. Moreover, both patients and healthcare professionals highlighted the potential benefit of greater public and professional awareness of the incidence of lung cancer in people who have never smoked on improving symptom reporting and increasing the likelihood of a more timely diagnosis [23,25].

### 3.3. Diagnosis

Both healthcare professionals and never smokers with lung cancer felt that delayed diagnosis was, to some extent, linked to both groups being less likely to attribute reported symptoms to lung cancer [23,24]. Healthcare professionals could be better supported to understand that persistent symptoms that would otherwise not warrant further investigation should be taken more seriously [24], and clinical guidance may need to be revised to reflect this [23]. Diagnosing lung cancer in never smokers is challenging in the context of clinicians’ usual risk-informed decision-making [23,25]. In the UK, the criteria for screening currently exclude this cohort [24]. Moreover, diagnostic guidance and risk models take into account smoking status when investigating suspected lung cancers. Unless never smokers are displaying particularly severe symptoms, the index of suspicion for lung cancer remains low. In addition, some healthcare professionals reported that they often did not ask individuals about their exposure to other risk factors, such as Radon or second-hand smoke. Instead, healthcare professionals often relied on “heuristics”, “rules of thumb”, or “gut feeling” based on weighing up patient health state and patient self-advocacy when making diagnostic decisions. It was noted that this approach differs based on specialty, with respiratory clinicians applying different heuristics to generalists as the latter are less experienced with lung cancer in never smokers. Those who have a specialty in respiratory disease will be more likely to have lung cancer in mind as a possible cause of symptoms, whereas general practitioners are more likely to rule it out based on the age and smoking status of the patient [25].

Delayed diagnosis in lung cancer patients who have never smoked is further complicated by the fact that they are more likely to report experiencing a single or no symptoms prior to diagnosis [23]. Moreover, never smokers often do not consider themselves at risk of lung cancer, making them more likely to attribute their symptoms to “seasonal illness” or “environmental factors” and less likely to access primary care [23]. Reticence by those who do not smoke to seek advice in primary care is supported by a survey of EGFR-, ALK-, and ROS1-positive patients in which almost half were diagnosed via emergency departments [29]. Moreover, when advice in primary care was sought, never smokers were found to be more likely to be reassured by their GP than smokers because they had “low levels of concern” about their symptoms [23].

### 3.4. Emotional Response

In addition to stigma, there was also evidence in the literature that never smokers have a different emotional response to a lung cancer diagnosis than those who have a history of smoking [20,21]. Diagnosis may be harder for this cohort because they are more likely to be fit and healthy, meaning it is likely the first and only significant health issue they have faced [21]. Participants reported a range of emotions, including anger, shock, and resentment [20,21]. Participants viewed the diagnosis as both unjust and undeserved due to a belief that they had done the right thing with regard to their own health, perceiving it almost as a punishment [20,21]. Participants viewed the diagnosis as both unjust and undeserved due to a belief that they done “all the right things” with regard to their own health [21]. Some perceived the diagnosis as a punishment given to them, despite having previously felt like a good person [20]. A sense of anger was also often explained as being a direct consequence of the apparent absence of causality [20]. Conversely, in a minority of cases, individuals found the lack of causality cathartic, freeing them to think more positively about treatment options, which was noted by the author as a potential area of further research [20].

### 3.5. Support

There is a paucity of evidence examining psychosocial and holistic support targeted at those who have never smoked but have been diagnosed with lung cancer, but the limited data available suggests that it is valued by this cohort [24,29]. Abbott et al. (2021) examined the benefits of support groups for individuals who were ALK-, EGFR-, or ROS1-positive [29]. The study explored access to support groups for individuals with these oncogenic driver mutations, and the majority of their participants had never smoked. The study highlighted the need for individualised support and explored what form that support may take. Most survey respondents found membership of a support group extremely (62.7%) or very (28.9%) valuable, providing a strong indication of the overall benefit of this kind of targeted support. Tailored support gave respondents access to information specific to their mutation rather than lung cancer generally, meaning that they felt *better prepared and informed* (79.6%) and received *better support* through membership (87.8%) [29].

Abbott et al. (2021) [29] also identified emotional benefits to being part of a support group more tailored to individual experiences. Participants were *inspired* by the experiences of other members of the group (71.3%) and felt that it had helped with *feelings of isolation* (49.7%), and the support group had provided them with a *safe place* (66.5%). Almost three-quarters of respondents reported that they would *recommend the group* (72.5%) to other patients. Moreover, no respondents agreed with the statement that *it has not helped me* (0%). Ultimately, the findings of this study confirmed the benefits to patients of focused support based on the underlying cause of their lung cancer [29].

## 4. Discussion

This integrative review has synthesised evidence from ten studies to explore commonalities and variations in the care pathways, support, and experiences of people with lung cancer who have never smoked. This has provided insight into key factors that are associated with individual experiences of different parts of the care pathway and how this is impacted by individual responses, perceived perceptions, levels of understanding and interactions with healthcare professionals, and the voluntary sector. This will allow for supportive care, including psychosocial care, education, and peer support, to be improved to better meet the needs of this patient cohort.

The concept of stigma in relation to a lung cancer diagnosis is defined broadly as an emotional response linked to misconceptions of lung cancer being a self-inflicted disease, provoking feelings of shame, guilt, and personal responsibility, either experienced internally or projected from others [12]. Lung cancer-related stigma has a far-reaching impact on patient behaviour and outcomes, including late diagnosis, delayed help-seeking, and increased levels of psychological distress, including anxiety, depression, and fear [12,30,31]. Individuals with suspected lung cancer are less likely to present with symptoms due to fear that they will be blamed for their illness or that they may not qualify for treatment as their condition is “self-inflicted” [12]. This is mirrored with other conditions associated with high levels of stigma, such as HIV and liver disease [9,10]. There is clear evidence that stigma is central to the experience of many patients diagnosed with lung cancer who have never smoked [20,21,22,24,26,27,28]. However, findings from the current review suggest that the experience of lung cancer-related stigma differs according to smoking status, with patients who have never smoked experiencing comparably less internalised stigma but still experiencing considerable perceived stigma, which they felt was impacting the care and support they received.

In addition, lung cancer patients have been found to have comparatively high levels of psychological distress, which can in part be explained by the experience of stigma but is also associated with demographic, social, illness, and treatment factors [32]. Diagnosis is a particularly vulnerable period for patients, with almost 70% of recently diagnosed cancer patients having high levels of anxiety, depression, and cancer-related fear [33]. Psychological distress in lung cancer patients is associated with lower quality of life, higher symptom burden, and poorer treatment outcomes [30,34]. Findings from the current review indicate that the emotional response to diagnosis is particularly severe for lung cancer patients who have never smoked because it is often so unexpected.

Addressing the experience of stigma in lung cancer patients and supporting them to manage the associated psychological distress should be a central component of the care pathway, as it has the potential to improve quality of life, treatment outcomes, and survival [35]. There is evidence that a range of interventions, including cognitive behavioural therapy, individual and group psychotherapy, psycho-education, exercise-based interventions, mindfulness-based programmes, and holistic supportive and palliative care, have the potential to help cancer patients manage the symptoms of psychological distress [36,37,38]. Moreover, interventions to reduce stigma, including educational, behavioural, and psychosocial interventions, have been evaluated positively in respiratory disease and across different cancer types more broadly. However, further research is required to determine the effectiveness of evidence-based interventions for lung cancer patients who have never smoked. Interventions aimed at reducing stigma in lung cancer and COPD are often effective at targeting internal stigma [39], yet further research is required to explore interventions that also counter external stigma, including individual interventions and public awareness campaigns.

Public awareness and education campaigns for lung cancer have traditionally focused on smoking cessation and can add to the stigma experienced by lung cancer patients [40]. A number of organisations and charities have run information campaigns in recent years promoting awareness of lung cancer in those who have never smoked [41,42]. Public awareness can lead to earlier presentation of patients to primary care, which may ultimately lead to earlier diagnosis [43]. What appears to be particularly challenging in lung cancer in those who have never smoked is that clinical guidance and risk models make the investigation and diagnosis of lung cancer in never smokers less salient, making late-stage diagnosis more likely and further compounding the issue [23]. Although the evidence is not clear, there are some suggestions that lung cancer in never smokers is diagnosed at a later stage than that of smokers, suggesting that survival rates could be improved if more timely diagnoses could be realised [13,44].

Education, information provision, and health literacy are associated with better patient outcomes, increased self-efficacy, and improvements in health-related quality of life [45]. Khan et al. (2023) suggest that an awareness campaign for both the public and healthcare professionals could be beneficial on the basis that a similar approach for lung cancer symptoms generally has been shown to lead to earlier diagnosis (Ironmonger et al., 2015; Kennedy et al., 2018) [24,46,47]. This is supported by Black et al. (2024), who raised concerns about the dismissal of patients in primary care who present with lung cancer symptoms if they did not smoke [25]. Increasing awareness of lung cancer in those who have never smoked has been identified in this review as having the potential to deal with some of the negative connotations of stigma, as well as having the potential to increase earlier diagnosis and help to ameliorate the negative emotional reaction to diagnosis.

There is a growing body of evidence to suggest that peer support is effective at supporting cancer patients to manage psychological symptoms of depression and anxiety, improve quality of life, and enhance overall well-being [48]. Peer support also has the potential to alleviate the burden on health services that are under-resourced and over-stretched [49]. This review reports on the overwhelming popularity of support groups tailored to those with lung cancer caused by a specific mutation. Indeed, tailored support for this cohort has also grown in prominence in recent years, with a substantial proportion being online [41,50]. The potential for online support to be beneficial to lung cancer patients has been highlighted, providing patients with the opportunity to exchange health information and establish networks of shared experience across geographic boundaries has been highlighted, although risks have also been identified [51]. For never smokers with lung cancer, it is unclear if support that extends beyond oncogene mutations is available or effective. Further examination of the content, delivery, and impact of the support that such groups provide is required and has the potential to identify gaps in support and inform the design of support going forward to ensure that any unmet needs can be addressed.

### Limitations and Strengths

The limited number of studies included in this review (*n* = 10) ultimately restricts the generalisability of the findings. This was a consequence of the lack of research conducted in this area, which was, to some extent, mitigated by the adoption of the integrative review methodology. This allowed for the inclusion of two studies that would not have met the inclusion criteria of a traditional systematic review methodology, broadening the scope of the review.

Despite searching the international literature, the findings were almost exclusively drawn from participants from the UK and the USA. Given that there are higher proportions of individuals diagnosed with lung cancer who have never smoked in other regions of the world, there is a concern that the findings may have limited applicability for global populations where the number of affected patients is higher.

Due to the heterogeneity of the extracted data, meta-analysis of the quantitative data was not possible, potentially limiting the precision of the review findings.

The present review identifies many of the issues associated with the care of lung cancer patients who have never smoked, as well as their support and information needs. However, it was not possible to assess the effectiveness of interventions that could be utilised to address these needs due to the nature of the studies included in the review. It was also not possible to draw inferences related to the demographic characteristics of the participants due to the way in which data were presented in the included studies.

## 5. Conclusions

There remains a paucity of literature exploring the care pathway, support, or experiences of individuals diagnosed with lung cancer who have never smoked. This review of the international literature has helped to identify areas where future research may be targeted. The stigma associated with a diagnosis of lung cancer is pervasive and can negatively impact this cohort, affecting diagnosis and the associated emotional experience. Increasing awareness and providing tailored support have the potential to have a positive effect, and more research in this area can help tailor and shape a different approach. Consideration of these factors will be crucial in developing targeted and effective psychological support to meet the needs of this patient group.

## Figures and Tables

**Table 1:** PubMed search strategy.

Population	“Never-smok*”[Title/Abstract] OR “Never smok*”[Title/Abstract] OR “Non-smok*”[Title/Abstract] OR “Non smok*”[Title/Abstract] OR “Nonsmok*”[Title/Abstract] OR “Don’t smok*”[Title/Abstract] OR “Never-Tobacco” [Title/Abstract] OR “Never Tobacco”[Title/Abstract] OR “Non-tobacco”[Title/Abstract] OR “Non tobacco”[Title/Abstract] OR “Nontobacco”[Title/Abstract] OR “Never-cigar*”[Title/Abstract] OR “Never cigar*”[Title/Abstract] OR “Passive smok*”[Title/Abstract] OR “Involuntary smok*”[Title/Abstract] OR “Second-hand smok*”[Title/Abstract] OR “Non-Smokers”[Mesh] OR “Tobacco Smoke Pollution”[Mesh]data
AND	
Exposure	“Lung cancer”[Title/Abstract] OR “Pulmonary cancer”[Title/Abstract] OR “Cancer of the Lung”[Title/Abstract] OR “Lung carcinoma”[Title/Abstract] OR “Pulmonary carcinoma”[Title/Abstract] OR “Carcinoma of the lung”[Title/Abstract] OR “Lung neoplasm”[Title/Abstract] OR “Pulmonary neoplasm”[Title/Abstract] OR “Small cell lung cancer”[Title/Abstract] OR “Non-small cell lung cancer”[Title/Abstract] OR “Lung neoplasm”[Title/Abstract] OR “ALK-positive”[Title/Abstract] OR “Anaplastic Lymphoma Kinase”[Title/Abstract] OR “lung tumor*”[Title/Abstract] OR “EGFR-positive”[Title/Abstract] OR “Epidermal Growth Factor Receptor”[Title/Abstract] OR “Lung Neoplasms”[Mesh] OR “Small Cell Lung Carcinoma”[Mesh] OR “Carcinoma, Non-Small-Cell Lung”[Mesh] OR “Anaplastic Lymphoma Kinase”[Mesh] OR “ErbB Receptors”[Mesh] data
AND	
Outcome	“Guidance”[Title/Abstract] OR “Support”[Title/Abstract] OR “Help”[Title/Abstract] OR “Assistance”[Title/Abstract] OR “Experience*”[Title/Abstract] OR “Impact”[Title/Abstract] OR “Burden”[Title/Abstract] OR “Care”[Title/Abstract] OR “Treatment*”[Title/Abstract] OR “Diagnos*”[Title/Abstract] OR “Pathway*”[Title/Abstract] OR “Screening”[Title/Abstract] OR “Early-diagnosi*”[Title/Abstract] OR “Presentation”[Title/Abstract] OR “Barrier*”[Title/Abstract] OR “Detection”[Title/Abstract] OR “Management”[Title/Abstract] OR “Therap*”[Title/Abstract] OR “Treatment Outcome”[Mesh] OR “Treatment Failure”[Mesh] OR “Treatment Delay”[Mesh] OR “Time-to-Treatment”[Mesh] OR “Critical Pathways”[Mesh] OR “Diagnosis”[Mesh] OR “Delayed Diagnosis”[Mesh] OR “Early Diagnosis”[Mesh] OR “Missed Diagnosis”[Mesh] OR “Early Detection of Cancer”[Mesh] OR “Health Impact Assessment”[Mesh] OR “Mass Screening”[Mesh] OR “Disease Management”[Mesh] OR “Disease Management”[Mesh]

**Table 2:** List of included studies.

Author (Year)	Country	Study Aims	Participants	Study Design	Key Findings/Outcomes
Abbott, Beattie, and Montague (2021) [29]	Worldwide (Charities based in the UK)	To provide insight into support groups for individuals with ALK+, EGFR+, and ROS1 lung cancer mutations.	167 lung cancer patients	Quantitative cross-sectional survey	Support groups are “extremely” or “very” valuable to most lung cancer patient participants (91.6%) with an oncogene mutation and represent a valuable tool in supporting them. More could be done through the groups to combat loneliness and help patients “advocate for better care”.
Black et al. (2022) [22]	UK	To explore the different diagnostic experiences of individuals diagnosed with lung cancer depending on smoking status.	40 lung cancer patients	Qualitative semi-structured interviews	Individuals who have never smoked are less vigilant about lung cancer symptoms and more likely to accept an initial alternative diagnosis.
Black et al. (2024) [23]	UK	To better understand how smoking history affects risk-informed decision-making in the lung cancer diagnostic pathway.	20 never smoker lung cancer patients; 10 clinicians	Qualitative semi-structured interviews	There is a need to improve awareness of lung cancer in never smokers amongst clinicians, and guidance should be updated to avoid delays to diagnosis caused by overreliance on smoking history as a marker.
Brandt (2015) [20]	USA	To explore the experiences of individuals diagnosed with lung cancer who have never smoked.	10 never smoker lung cancer patients	Qualitative phenomenological, unstructured interviews	The experience of lung cancer in never smokers is uniquely challenging, emotionally complex, and under-recognised in both research and clinical care.
Criswell et al. (2016) [26]	USA	To explore rates and intensity of regret, personal responsibility, and medical stigma amongst individuals with a lung cancer diagnosis with different smoking histories, and to measure their impact on psychosocial and health-related outcomes.	213 lung cancer patients	Quantitative cross-sectional survey	Ever and current smokers experienced significantly higher levels of regret and personal responsibility than never smokers, but all groups experienced medical stigma to a similar degree. Associations between all factors of the Cancer Responsibility and Regret Scale and depressive symptoms were significantly higher in never smokers, suggesting that this group could be more adversely affected by regret, personal responsibility, and medical stigma.
Dao et al. (2019) [21]	USA	To explore the consequences of stigma from the perspective of non-smoking women with lung cancer, with a focus on healthcare, to better understand how this information can influence providers.	23 lung cancer patients	Qualitative semi-structured interviews	The diagnosis of lung cancer was emotionally difficult, and individuals felt that the diagnosis of their cancer had been delayed as a result of their smoking status.
Khan et al. (2023) [24]	UK	To determine the areas in need of further study to promote better outcomes for individuals with lung cancer who have never smoked tobacco.	127 stakeholder survey participants; 190 stakeholders took part in consensus work	Qualitative cross-sectional survey and consensus work	Areas of concern for further research included delayed diagnosis, the need to increase knowledge of lung cancer in never smokers, the need to increase awareness of other causes beyond smoking, and further research into the care pathway. There is a need to tackle the stigma attached to a diagnosis.
*Lung Cancer Europe* (2022) [22]	Europe	To explore lung cancer patients’ experiences of the care pathway, from diagnosis to treatment and follow-up.	991 lung cancer patients	Qualitative cross-sectional survey	There is a need to speed up the diagnostics process, provide more support for patients on the treatment pathway, and improve supportive care for all lung cancer patients. There is a suggestion that never smokers experience each of these differently, but there is limited data as to how.
Williamson et al. (2018) [27]	USA	To explore the association between internalised stigma and constrained disclosure, with quality of life and associated physical and mental well-being.	101 lung cancer patients	Quantitative longitudinal survey	Never smokers reported less internalised stigma than former smokers (*p* = 0.013) and current smokers (*p* = 0.001). There was a significant association between higher levels of internalised stigma and declining emotional well-being at 6 (*p* = 0.002 and 12 weeks (*p* = 0.004).
Williamson et al. (2020) [28]	USA	To discover if there is a relationship between smoking status and lung cancer stigma amongst patients, and if there is a link with depressive symptoms.	266 lung cancer patients	Quantitative cross-sectional survey	Current smokers scored significantly more for total lung cancer stigma than former smokers (*p* = 0.003) and never smokers (*p* < 0.001). Lung cancer stigma scores are strongly associated with higher depressive symptoms, irrespective of smoking status (all *p* < 0.001).

## Data Availability

No new data were created or analyzed in this study. Data sharing is not applicable to this article.

## Supplementary Materials

The following supporting information can be downloaded at https://www.mdpi.com/article/10.3390/curroncol33010004/s1, File S1: Search Strategy; File S2: Quality Appraisal; File S3: PRISMA Checklist. References [16,18,19] are cited in the Supplementary Materials.
