# Supplementary material for: An Integrative Review to Examine the Care Pathways and Support Available for Individuals Diagnosed with Lung Cancer Who Have Never Smoked
Source: Curr Oncol. 2025 Dec 20;33(1):4. doi: 10.3390/curroncol33010004 (PMC12839640; doi:10.3390/curroncol33010004)
Supplement: Supplementary file 1 [file curroncol-33-00004-s001.zip › Supplementary Materials File S1. Search Strategy.pdf]

An integrative review to examine the care pathways and sup-port available for individuals diagnosed with lung cancer who have never smoked
Dodd, C., Henshall, C., Jain, M., & Davey, Z.

Supplemental Material

Search Strategy

PubMed

Population

"Never-smok*"[Title/Abstract] OR "Never smok*"[Title/Abstract] OR "Non-smok*"[Title/Abstract] OR "Non smok*"[Title/Abstract] OR "Nonsmok*"[Title/Abstract] OR "Don’t smok*"[Title/Abstract] OR "Never-Tobacco" [Title/Abstract] OR "Never Tobacco"[Title/Abstract] OR "Non-tobacco"[Title/Abstract] OR "Non tobacco"[Title/Abstract] OR "Nontobacco"[Title/Abstract] OR "Never-cigar*"[Title/Abstract] OR "Never cigar*"[Title/Abstract] OR "Passive smok*"[Title/Abstract] OR "Involuntary smok*"[Title/Abstract] OR "Second-hand smok*"[Title/Abstract] OR "Non-Smokers"[Mesh] OR "Tobacco Smoke Pollution"[Mesh]

AND

Exposure

"Lung cancer"[Title/Abstract] OR "Pulmonary cancer"[Title/Abstract] OR "Cancer of the Lung"[Title/Abstract] OR "Lung carcinoma"[Title/Abstract] OR "Pulmonary carcinoma"[Title/Abstract] OR "Carcinoma of the lung"[Title/Abstract] OR "Lung neoplasm"[Title/Abstract] OR "Pulmonary neoplasm"[Title/Abstract] OR "Small cell lung cancer"[Title/Abstract] OR "Non-small cell lung cancer"[Title/Abstract] OR "Lung neoplasm"[Title/Abstract] OR "ALK-positive"[Title/Abstract] OR "Anaplastic Lymphoma Kinase"[Title/Abstract] OR "lung tumor*"[Title/Abstract] OR "EGFR-positive"[Title/Abstract] OR "Epidermal Growth Factor Receptor"[Title/Abstract] OR "Lung Neoplasms"[Mesh] OR "Small Cell Lung Carcinoma"[Mesh] OR "Carcinoma, Non-Small-Cell Lung"[Mesh] OR "Anaplastic Lymphoma Kinase"[Mesh] OR "ErbB Receptors"[Mesh]

AND

Outcome

"Guidance"[Title/Abstract] OR "Support"[Title/Abstract] OR "Help"[Title/Abstract] OR "Assistance"[Title/Abstract] OR "Experience*"[Title/Abstract] OR "Impact"[Title/Abstract] OR "Burden"[Title/Abstract] OR "Care"[Title/Abstract] OR "Treatment*"[Title/Abstract] OR "Diagnos*"[Title/Abstract] OR "Pathway*"[Title/Abstract] OR "Screening"[Title/Abstract] OR

An integrative review to examine the care pathways and sup-port available for individuals diagnosed with lung cancer who have never smoked
Dodd, C., Henshall, C., Jain, M., & Davey, Z.

Supplemental Material

"Early-diagnosi*"[Title/Abstract] OR "Presentation"[Title/Abstract] OR "Barrier*"[Title/Abstract] OR "Detection"[Title/Abstract] OR "Management"[Title/Abstract] OR "Therap*"[Title/Abstract] OR "Treatment Outcome"[Mesh] OR "Treatment Failure"[Mesh] OR "Treatment Delay"[Mesh] OR "Time-to-Treatment"[Mesh] OR "Critical Pathways"[Mesh] OR "Diagnosis"[Mesh] OR "Delayed Diagnosis"[Mesh] OR "Early Diagnosis"[Mesh] OR "Missed Diagnosis"[Mesh] OR "Early Detection of Cancer"[Mesh] OR "Health Impact Assessment"[Mesh] OR "Mass Screening"[Mesh] OR "Disease Management"[Mesh] OR "Disease Management"[Mesh]

An integrative review to examine the care pathways and sup-port available for individuals diagnosed with lung cancer who have never smoked
Dodd, C., Henshall, C., Jain, M., & Davey, Z.

Supplemental Material

Web of Science

Population

(AB=("Never-smok*" OR "Never smok*" OR "Non-smok*" OR "Non smok*" OR "Nonsmok*" OR "Don’t smok*" OR "Never-Tobacco" OR "Never Tobacco" OR "Non-tobacco" OR "Non tobacco" OR "Nontobacco" OR "Never-cigar*" OR "Never cigar*" OR "Passive smok*" OR "Involuntary smok*" OR "Second-hand smok*")) OR (TI=("Never-smok*" OR "Never smok*" OR "Non-smok*" OR "Non smok*" OR "Nonsmok*" OR "Don’t smok*" OR "Never-Tobacco" OR "Never Tobacco" OR "Non-tobacco" OR "Non tobacco" OR "Nontobacco" OR "Never-cigar*" OR "Never cigar*" OR "Passive smok*" OR "Involuntary smok*" OR "Second-hand smok*"))

AND

Exposure

(AB=("Lung cancer" OR "Pulmonary cancer" OR "Cancer of the Lung" OR "Lung carcinoma" OR "Pulmonary carcinoma" OR "Carcinoma of the lung" OR "Lung neoplasm" OR "Pulmonary neoplasm" OR "Small cell lung cancer" OR "Non-small cell lung cancer" OR "Lung neoplasm" OR "ALK-positive" OR "Anaplastic Lymphoma Kinase" OR "lung tumor*" OR "EGFR-positive" OR "Epidermal Growth Factor Receptor")) OR (TI=("Lung cancer" OR "Pulmonary cancer" OR "Cancer of the Lung" OR "Lung carcinoma" OR "Pulmonary carcinoma" OR "Carcinoma of the lung" OR "Lung neoplasm" OR "Pulmonary neoplasm" OR "Small cell lung cancer" OR "Non-small cell lung cancer" OR "Lung neoplasm" OR "ALK-positive" OR "Anaplastic Lymphoma Kinase" OR "lung tumor*" OR "EGFR-positive" OR "Epidermal Growth Factor Receptor"))

AND

Outcome

(AB=("Guidance" OR "Support" OR "Help" OR "Assistance" OR "Experience*" OR "Impact" OR "Burden" OR "Care" OR "Treatment*" OR "Diagnos*" OR "Pathway*" OR "Screening" OR "Early-diagnosi*" OR "Presentation" OR "Barrier*" OR "Detection" OR "Management" OR "Therap*")) OR (TI=("Guidance" OR "Support" OR "Help" OR "Assistance" OR "Experience*" OR "Impact" OR "Burden" OR "Care" OR "Treatment*" OR "Diagnos*" OR

An integrative review to examine the care pathways and sup-port available for individuals diagnosed with lung cancer who have never smoked
Dodd, C., Henshall, C., Jain, M., & Davey, Z.

Supplemental Material

"Pathway*" OR "Screening" OR "Early-diagnosi*" OR "Presentation" OR "Barrier*" OR "Detection" OR "Management" OR "Therap*"))

An integrative review to examine the care pathways and sup-port available for individuals diagnosed with lung cancer who have never smoked
Dodd, C., Henshall, C., Jain, M., & Davey, Z.

Supplemental Material

CINAHL

Population

(AB ("Never-smok*" OR "Never smok*" OR "Non-smok*" OR "Non smok*" OR "Nonsmok*" OR "Don’t smok*" OR "Never-Tobacco" OR "Never Tobacco" OR "Non-tobacco" OR "Non tobacco" OR "Nontobacco" OR "Never-cigar*" OR "Never cigar*" OR "Passive smok*" OR "Involuntary smok*" OR "Second-hand smok*")) OR (TI ("Never-smok*" OR "Never smok*" OR "Non-smok*" OR "Non smok*" OR "Nonsmok*" OR "Don’t smok*" OR "Never-Tobacco" OR "Never Tobacco" OR "Non-tobacco" OR "Non tobacco" OR "Nontobacco" OR "Never-cigar*" OR "Never cigar*" OR "Passive smok*" OR "Involuntary smok*" OR "Second-hand smok*")) OR (MH ("Non-Smokers" OR "Passive Smoking"))

AND

Exposure

(AB ("Lung cancer" OR "Pulmonary cancer" OR "Cancer of the Lung" OR "Lung carcinoma" OR "Pulmonary carcinoma" OR "Carcinoma of the lung" OR "Lung neoplasm" OR "Pulmonary neoplasm" OR "Small cell lung cancer" OR "Non-small cell lung cancer" OR "Lung neoplasm" OR "ALK-positive" OR "Anaplastic Lymphoma Kinase" OR "lung tumor*" OR "EGFR-positive" OR "Epidermal Growth Factor Receptor")) OR (TI ("Lung cancer" OR "Pulmonary cancer" OR "Cancer of the Lung" OR "Lung carcinoma" OR "Pulmonary carcinoma" OR "Carcinoma of the lung" OR "Lung neoplasm" OR "Pulmonary neoplasm" OR "Small cell lung cancer" OR "Non-small cell lung cancer" OR "Lung neoplasm" OR "ALK-positive" OR "Anaplastic Lymphoma Kinase" OR "lung tumor*" OR "EGFR-positive" OR "Epidermal Growth Factor Receptor")) OR (MH "Carcinoma, Non-Small-Cell Lung" OR "Lung Neoplasms" OR "Carcinoma, Small Cell" OR "Anaplastic Lymphoma Kinase" OR "Epidermal Growth Factors"))

AND

Outcome

(AB ("Guidance" OR "Support" OR "Help" OR "Assistance" OR "Experience*" OR "Impact" OR "Burden" OR "Care" OR "Treatment*" OR "Diagnos*" OR "Pathway*" OR "Screening" OR "Early-diagnosi*" OR "Presentation" OR "Barrier*" OR "Detection" OR "Management"

OR "Therap*")) OR (TI ("Guidance" OR "Support" OR "Help" OR "Assistance" OR "Experience*" OR "Impact" OR "Burden" OR "Care" OR "Treatment*" OR "Diagnos*" OR "Pathway*" OR "Screening" OR "Early-diagnosi*" OR "Presentation" OR "Barrier*" OR "Detection" OR "Management" OR "Therap*"))

An integrative review to examine the care pathways and sup-port available for individuals diagnosed with lung cancer who have never smoked
Dodd, C., Henshall, C., Jain, M., & Davey, Z.

Supplemental Material

Psychinfo

Population

(AB ("Never-smok*" OR "Never smok*" OR "Non-smok*" OR "Non smok*" OR "Nonsmok*" OR "Don’t smok*" OR "Never-Tobacco" OR "Never Tobacco" OR "Non-tobacco" OR "Non tobacco" OR "Nontobacco" OR "Never-cigar*" OR "Never cigar*" OR "Passive smok*" OR "Involuntary smok*" OR "Second-hand smok*")) OR (TI ("Never-smok*" OR "Never smok*" OR "Non-smok*" OR "Non smok*" OR "Nonsmok*" OR "Don’t smok*" OR "Never-Tobacco" OR "Never Tobacco" OR "Non-tobacco" OR "Non tobacco" OR "Nontobacco" OR "Never-cigar*" OR "Never cigar*" OR "Passive smok*" OR "Involuntary smok*" OR "Second-hand smok*")) OR (DE "Passive Smoking")

AND

Exposure

(AB ("Lung cancer" OR "Pulmonary cancer" OR "Cancer of the Lung" OR "Lung carcinoma" OR "Pulmonary carcinoma" OR "Carcinoma of the lung" OR "Lung neoplasm" OR "Pulmonary neoplasm" OR "Small cell lung cancer" OR "Non-small cell lung cancer" OR "Lung neoplasm" OR "ALK-positive" OR "Anaplastic Lymphoma Kinase" OR "lung tumor*" OR "EGFR-positive" OR "Epidermal Growth Factor Receptor")) OR (TI ("Lung cancer" OR "Pulmonary cancer" OR "Cancer of the Lung" OR "Lung carcinoma" OR "Pulmonary carcinoma" OR "Carcinoma of the lung" OR "Lung neoplasm" OR "Pulmonary neoplasm" OR "Small cell lung cancer" OR "Non-small cell lung cancer" OR "Lung neoplasm" OR "ALK-positive" OR "Anaplastic Lymphoma Kinase" OR "lung tumor*" OR "EGFR-positive" OR "Epidermal Growth Factor Receptor")) OR (DE "Lung Neoplasms")

AND

Outcome

(AB ("Guidance" OR "Support" OR "Help" OR "Assistance" OR "Experience*" OR "Impact" OR "Burden" OR "Care" OR "Treatment*" OR "Diagnos*" OR "Pathway*" OR "Screening" OR "Early-diagnosi*" OR "Presentation" OR "Barrier*" OR "Detection" OR "Management" OR "Therap*")) OR (TI ("Guidance" OR "Support" OR "Help" OR "Assistance" OR "Experience*" OR "Impact" OR "Burden" OR "Care" OR "Treatment*" OR "Diagnos*" OR

An integrative review to examine the care pathways and sup-port available for individuals diagnosed with lung cancer who have never smoked

Dodd, C., Henshall, C., Jain, M., & Davey, Z.

Supplemental Material

"Pathway*" OR "Screening" OR "Early-diagnosi*" OR "Presentation" OR "Barrier*" OR "Detection" OR "Management" OR "Therap*"))
