# Supplementary material for: An Integrative Review to Examine the Care Pathways and Support Available for Individuals Diagnosed with Lung Cancer Who Have Never Smoked
Source: Curr Oncol. 2025 Dec 20;33(1):4. doi: 10.3390/curroncol33010004 (PMC12839640; doi:10.3390/curroncol33010004)
Supplement: Supplementary file 1 [file curroncol-33-00004-s001.zip › Supplementary Materials File S2. Quality Appraisal.pdf]

An integrative review to examine the care pathways and sup-port available for individuals diagnosed with lung cancer who have never smoked

Dodd, C., Henshall, C., Jain, M., & Davey, Z.

Supplemental Material

Quality appraisal

Qualitative studies

**Author (Year)**	**Is there congruity between the stated philosophical perspective and the research methodology?**	**Is there congruity between the research methodology and the research question or objectives?**	**Is there congruity between the research methodology and the methods used to collect data?**	**Is there congruity between the research methodology and the representation and analysis of data?**	**Is there congruity between the research methodology and the interpretation of results?**	**Is there a statement locating the researcher culturally or theoretically?**	**Is the influence of the researcher on the research, and vice-versa, addressed?**	**Are participants, and their voices, adequately represented?**	**Is the research ethical according to current criteria or, for recent studies, and is there evidence of ethical approval by an appropriate body?**	**Do the conclusions drawn in the research report flow from the analysis, or interpretation, of the data?**
Lung Cancer Europe (2022)	✓	✓	✓	✓	✓	✓	✓	✓	X	✓
Black et al. (2022)	✓	✓	✓	✓	✓	✓	✓	✓	✓	✓
Black et al. (2024)	✓	✓	✓	✓	✓	✓	X	✓	✓	✓
**Author (Year)**	Is there congruity	Is there congruity	Is there congruity	Is there congruity	Is there congruity	Is there a statement	Is the influence of	Are participants,	Is the research	Do the conclusions

An integrative review to examine the care pathways and sup-port available for individuals diagnosed with lung cancer who have never smoked

Dodd, C., Henshall, C., Jain, M., & Davey, Z.

Supplemental Material

	between the stated philosophical perspective and the research methodology ?	between the research methodology and the research question or objectives?	between the research methodology and the methods used to collect data?	between the research methodology and the representatio n and analysis of data?	between the research methodology and the interpretation of results?	locating the researcher culturally or theoretically ?	the researcher on the research, and vice-versa, addressed?	and their voices, adequately represented ?	ethical according to current criteria or, for recent studies, and is there evidence of ethical approval by an appropriate body?	drawn in the research report flow from the analysis, or interpretation , of the data?
Brandt (2015)	✓	✓	✓	✓	✓	✓	✓	✓	✓	✓
Dao et al., (2019)	✓	✓	✓	✓	✓	✓	✓	✓	✓	✓
Khan et al. (2023)	✓	✓	✓	✓	✓	✓	n/a	✓	✓	✓

Lockwood C, Munn Z, Porritt K. Qualitative research synthesis: methodological guidance for systematic reviewers utilizing meta-aggregation. Int J Evid Based Healthc. 2015;13(3):179–187.

An integrative review to examine the care pathways and sup-port available for individuals diagnosed with lung cancer who have never smoked

Dodd, C., Henshall, C., Jain, M., & Davey, Z.

Supplemental Material

Quantitative studies

Author (Year	Were the criteria for inclusion in the sample clearly defined?	Were the study subjects and the setting described in detail?	Was the exposure measured in a valid and reliable way?	Were objective, standard criteria used for measurement of the condition?	Were confounding factors identified?	ere strategies to deal with confounding factors stated?	Were the outcomes measured in a valid and reliable way?	Was appropriate statistical analysis used?
Abbott, Beattie and Montague (2021)	✓	✓	n/a	✓	✓	✓	✓	✓
Criswell et al. (2016)	✓	✓	n/a	✓	✓	✓	✓	✓
Williamson et al. (2018)	✓	✓	n/a	✓	✓	✓	✓	✓
Williamson et al. (2020)	✓	✓	n/a	✓	✓	✓	✓	✓

Moola S, Munn Z, Tufanaru C, Aromataris E, Sears K, Sfetcu R, Currie M, Qureshi R, Mattis P, Lisy K, Mu P-F. Chapter 7: Systematic reviews of etiology and risk. In: Aromataris E, Munn Z (Editors). *JBI Manual for Evidence Synthesis.* JBI, 2020.
